# Therapeutic Applications of Resveratrol in Hepatic Encephalopathy through Its Regulation of the Microbiota, Brain Edema, and Inflammation

**DOI:** 10.3390/jcm10173819

**Published:** 2021-08-25

**Authors:** Young-Kook Kim, Juhyun Song

**Affiliations:** 1 Department of Biochemistry, Chonnam National University Medical School, Hwasun 58128, Jeollanam-do, Korea; ykk@chonnam.ac.kr; 2 Department of Anatomy, Chonnam National University Medical School, Hwasun 58128, Jeollanam-do, Korea

**Keywords:** hepatic encephalopathy, resveratrol, gut microbiota, brain edema, neuroinflammation

## Abstract

Hepatic encephalopathy is a common complication in patients with liver cirrhosis and portosystemic shunting. Patients with hepatic encephalopathy present a variety of clinical features, including neuropsychiatric manifestations, cognitive dysfunction, impaired gut barrier function, hyperammonemia, and chronic neuroinflammation. These pathogeneses have been linked to various factors, including ammonia-induced oxidative stress, neuronal cell death, alterations in the gut microbiome, astrocyte swelling, and blood-brain barrier disruptions. Many researchers have focused on identifying novel therapeutics and prebiotics in the hope of improving the treatment of these conditions. Resveratrol is a natural polyphenic compound and is known to exert several pharmacological effects, including antioxidant, anti-inflammatory, and neuroprotective activities. Recent studies suggest that resveratrol contributes to improving the neuropathogenic effects of liver failure. Here, we review the current evidence describing resveratrol’s effects in neuropathogenesis and its impact on the gut-liver axis relating to hepatic encephalopathy. We highlight the hypothesis that resveratrol exerts diverse effects in hepatic encephalopathy and suggest that these effects are likely mediated by changes to the gut microbiota, brain edema, and neuroinflammation.

## 1. Introduction

Hepatic encephalopathy (HE) is a type of liver failure affecting up to 40% of all liver cirrhosis patients [1], and epidemiological studies have shown that the prevalence of HE is gradually increasing all over the world [2,3]. A recent study identified HE as an impairment of the brain function caused by liver failure and portosystemic blood shunting, characterized by emotional impairment, cognitive dysfunction, psychiatric problems, and neuromuscular dysfunction [4,5]. Moreover, the patients with HE present impaired visual temporal perception [6] and impaired tactile temporal discrimination [7].

HE pathogenesis is linked to poor glucose utilization, impaired cerebral energy metabolism, mitochondrial dysfunction, oxidative stress, and high levels of ammonia [8,9]. Clinically, HE is mainly divided into overt HE and minimal HE [10]. Overt HE patients can be diagnosed through several symptoms and are present in almost 30% of patients with liver cirrhosis, whereas minimal HE patients can be diagnosed based on impairment in specialized tests and are considered as patients of a preclinical stage of overt HE [10,11]. Over 60% of patients with minimal HE suffer from cognitive dysfunction, which leads to poor life quality [10].

Although many researchers have tried to identify natural products with some therapeutic effect on HE to reduce the therapeutic side effects, there are still no approved natural compounds for the treatment of this condition.

Resveratrol (3,5,4′-trihydroxy-trans-stilbene) is a natural antioxidant polyphenol and is synthesized by various plants, including peanuts, berries, and grapes [12,13]. Resveratrol has been reported to exert anti-inflammatory, antiviral, and antioxidant effects in cells and has been shown to reduce oxidative stress-related cell damage [14,15,16]. Resveratrol acts as a reactive oxygen species (ROS) scavenger during oxidative stress and boosts the antioxidant enzyme activity [17]. Resveratrol regulates multiple cellular activities via its interactions with silent mating type information regulation 2 homolog 1 (SIRT1) [18]. SIRTs have been shown to be affected by resveratrol and are known to have a relationship with cellular energy metabolism, mitochondrial function, and cellular longevity [19], and a neuroprotective response [20]. One study observed that the treatment of embryonic stem cells with resveratrol resulted in an improved DNA repair when faced with DNA-damaging conditions [21], suggesting the potential of resveratrol to protect the tissue against ionizing radiation-induced damage [22]. In the brain, the expression of SIRT1 is widely observed in diverse neuronal nuclei and is commonly found in glia, neural stem cells, mature neuron [23], hypothalamus related with mood [24], and a suprachiasmatic nucleus related with sleep pattern [25]. A recent study mentioned that resveratrol-mediated SIRT1 activation reduces apical dendrite damage in hippocampal pyramidal neurons and enhances neurobehavioral impairment in HE rats [26].

In the central nervous system (CNS), resveratrol has been shown to exert neuroprotective effects in neurodegenerative disease models such as dementia [27] and depression [15,28]. Resveratrol, which is a lipophilic compound, crosses the blood-brain barrier (BBB) and enters the brain after intraperitoneal injection [29,30], ultimately influencing various neurological mechanisms within these tissues [31]. The resveratrol-mediated activation of the *SIRT1* gene induces increased ROS scavenging and ultimately improves cognitive function [32,33]. Although a meta-analysis study suggested that resveratrol has no significant effect on cognitive function [34], another meta-analysis study indicated that oral resveratrol treatment improves some cognitive performances in subjects [35]. Based on these reports, the effect of resveratrol on cognitive improvement has so far been controversial [36].

Here, we review significant pieces of evidence relating to the therapeutic effects of resveratrol in HE. We summarize the therapeutic potential of resveratrol in HE from several points of view, including its impact on the microbiota, brain edema, and inflammation.

## 2. Resveratrol and HE

**Figure 1:**
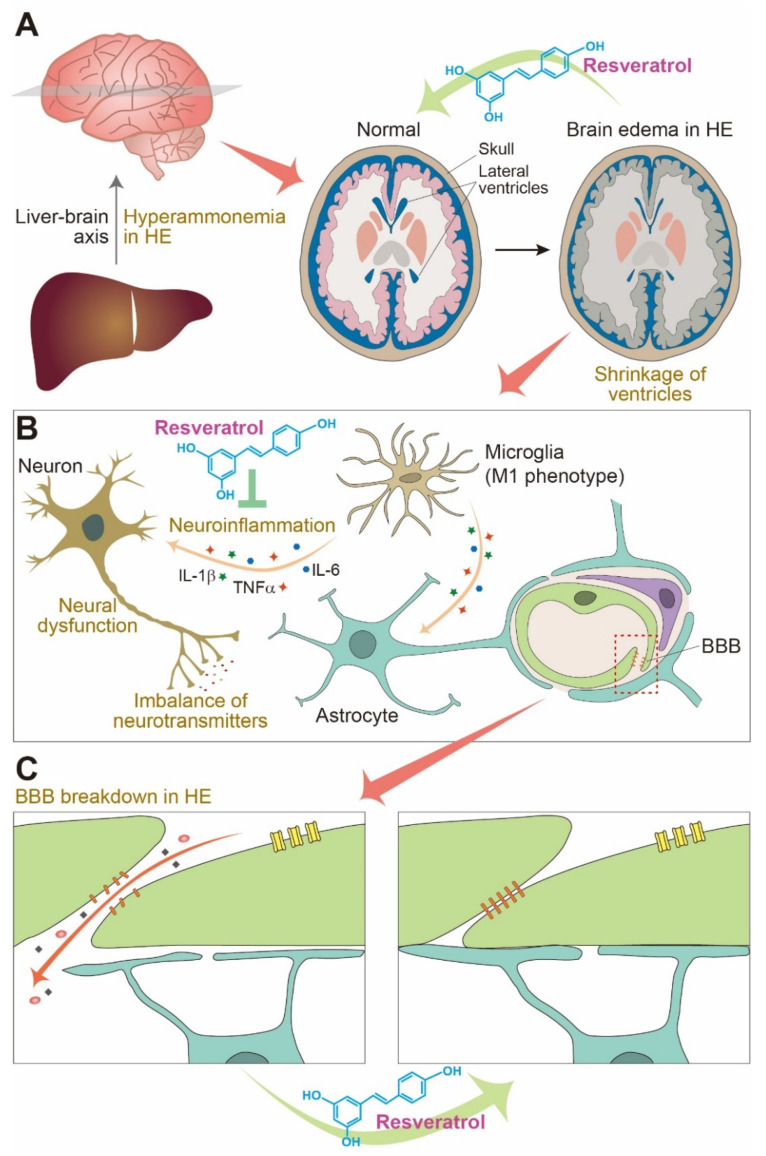
The pathogenic process of hepatic encephalopathy (HE) in the brain and the possible protective effects of resveratrol. (**A**) HE in the brain is linked to the development of several specific features including hyperammonemia, brain edema, neuroinflammation, and blood-brain barrier (BBB). Resveratrol may protect brain endothelial cells, astrocytes, and neurons against hyperammonemia-induced damage, and finally circumvents brain edema. (**B**) In addition, resveratrol may reduce neuroinflammation by suppressing inflammatory cytokine expression, and (**C**) protects against BBB disruption by helping to maintain a tight junction protein density within the BBB. Note that the protective effects of resveratrol shown in this figure are hypothetic effects based on the current empirical evidence and do not represent the proven effects of resveratrol on patients with HE. See text for details. IL: interleukin, TNF: tumor necrosis factor.

HE is a metabolic brain disorder associated with progressive liver failure and is characterized by neurological problems including brain edema which result in a variety of cognitive dysfunctions, such as attention deficit, motor dysfunction, memory impairment, emotional problems [37,38,39]. HE pathology is commonly reported in patients with liver cirrhosis and transjugular intrahepatic portosystemic shunts [40,41]. HE demonstrates several central features, including elevated levels of ammonia in the circulation and brain tissues, often referred to as hyperammonemia [42]. This hyperammonemia is a direct result of the disruption of the ammonia metabolism in the diseased liver [43,44]. HE has also been linked to imbalances in excitatory and inhibitory neurotransmitters such as GABA and glutamate and to the inactivation of neurotransmitter receptors [45,46]. In addition, HE leads to severe neuroinflammation, glial activation, and glial polarization in the brain, triggering increased oxidative stress [47]. HE aggravates neuronal dysfunction, inhibits the interactions between the neurons and glia [48,49], and may disrupt the BBB and induce cerebral edema [50,51,52] (Figure 1B,C).

Resveratrol is one of the polyphenols produced by berries and grapes [13] and has been known to exert several different cellular effects [15,16]. The addition of resveratrol has been reported to reduce the proliferation of liver myofibroblasts [53] and inhibit the over-accumulation of triacylglycerols through the activation of the AMP-activated protein kinase (AMPK) pathway in the liver cancer cell line [54]. It has also been reported that resveratrol treatment suppresses the growth of hepatic stellate cells [55] and induces the apoptosis of hepatic cancer cells [56]. Other studies have shown that resveratrol blocked the hydroquinone-induced cellular apoptosis of primary hepatocytes [57] and inhibited cellular oxidative stress responses in hepatocytes via its activation of catalase and glutathione peroxidase in these cells [58].

Resveratrol reverses the ethanol-induced impairment of energy homeostasis in the liver by increasing the ATP production in cellular mitochondria [59]. One study suggests that a resveratrol injection contributes to a normal liver function in liver-transplanted rats [60]. Another study reported that resveratrol improves hepatic glucose metabolism and insulin activity in the liver through the activation of several signaling pathways, including insulin receptor substrate 1 signaling, AKT phosphorylation signaling, and the peroxisome proliferating activation receptor-γ coactivator 1α pathway [61]. In addition, resveratrol supplementation reduced lipid peroxidation and increased the antioxidant enzyme activity in the liver [62], and a previous study mentioned that resveratrol treatment prevents cholestatic liver injury and induces hepatic regeneration after bile duct ligation [63]. One experimental study indicated that resveratrol reduces the amount of superoxide anions and the expression of inflammatory mediators while increasing antioxidant enzymes in response to lipopolysaccharide-induced hepatotoxicity [64].

Taken together, these reports suggest that resveratrol improves various liver dysfunctions. Although there are many types of liver failure, the discovery of efficient HE treatments has been particularly difficult because the onset and progression of HE are broad, systemic, and gradual, and largely facilitated by the brain–liver axis. This means that it is critical to identify an effective treatment for HE, and the current data suggest that resveratrol may be a promising candidate for these therapeutic interventions. Below, we discuss this point with a focus on the effects of resveratrol on the gut microbiota, brain edema, and neuroinflammation associated with HE.

## 3. Resveratrol and the Microbiome in HE

There are over 100 quintillion microorganisms in the gut, including bacteria and viruses, and its epithelium is considered the primary immune barrier against bacterial toxins [65,66]. The gut microbiome is a complicated system with a mass of approximately 1 kg per person [67]. In general, the human gut microbiota is divided into four main categories, including *Firmicutes*, *Actinobacteria*, *Bacteroidetes*, and *Proteobacteria* [68]. The gut microbiota act as protectants against pathogens and maintain a healthy immune homeostasis while also helping to facilitate digestion [69,70,71].

The gut and liver are connected via the portal vein, biliary tract, and systemic blood circulation [72]. Abnormal gut microbiota influence liver function via their connected network [73]. Patients with liver cirrhosis have presented a reduction in the expression of genes associated with the metabolism of various nutrients, such as amino acids and nucleotides [74]. Endotoxins and toxic molecules from the gut are transferred to the portal vein and may ultimately trigger the activation of inflammatory signaling in the liver [67]. Plasma endotoxin levels are increased in association with the progression of liver cirrhosis [75].

**Figure 2:**
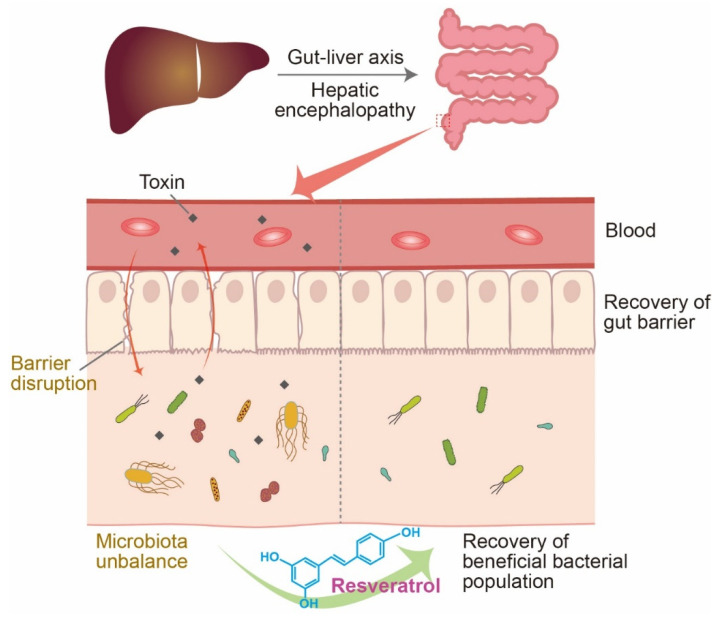
The pathogenesis of hepatic encephalopathy (HE) in the gut and possible therapeutic effects of resveratrol treatment. The gut microbiome is altered in response to hepatic encephalopathy, which increases the amount of toxins from harmful microorganisms within the system, exacerbating the pathogenesis of HE. HE reduces the integrity of the gut barrier, allowing for a systemic distribution of these toxic compounds, and increases inflammation and systemic disruption. However, the treatment with resveratrol may facilitate the recovery of the beneficial bacterial populations and improve the integrity of the gut barrier. It is also noted that the therapeutic effect of resveratrol shown in this figure is an assumption based on the current empirical evidence and not proven in patients with HE. See text for details.

Breakdowns in the gut barrier function contribute to liver failure by worsening the intestinal mucosal damage [76,77]. Dysbiosis, described by changes in the microbial community, is often caused by changes in the number and composition of the microbial communities in the gut [78]. Dysbiosis results in gut barrier impairment through the inactivation of the epithelial mucosal repair system and is common to a variety of inflammatory diseases [78,79]. The alteration of the gut microbiome is known to be a critical component in the pathogenesis and progression of liver cirrhosis, hepato-renal diseases, and HE [80,81] (Figure 2).

Several studies have demonstrated that the gut microbiome in liver cirrhosis patients presents with a decreased abundance of autochthonous bacteria such as *Subdoligranulum* and an increased abundance of pathogenic bacteria such as *Enterococcus*, *Dialister*, and *Prevotella* compared to the normal gut [82,83]. In addition, a recent study mentioned that the increase in ammonia accumulation associated with HE is strongly linked to the imbalances in the gut microbiota of these patients [84]. Interestingly, one study suggested that specific microbiota-based biomarkers could be used as a diagnostic factor for liver cirrhosis [85].

Probiotics, prebiotics, and natural compounds could promote the growth of beneficial microbes and the reduction of harmful microbes [86] in the gut of patients with liver failure [87,88,89,90]. One recent study tried to use a mixture of probiotics to treat HE pathogenesis by improving the health of the gut microbiota [91]. Several HE studies have shown that there are a number of probiotics and prebiotics, including lactulose, which can modulate the gut microbiota by reducing the intraluminal pH and decreasing the ammonia concentration, leading to improvements in the gut epithelium [92,93,94].

Resveratrol can modulate the gut microbiota by decreasing the levels of *Bacteroides*, *Alistipes, Odoribacter*, and *Parabacteroides* and improving the gut epithelial protection against the metabolic imbalances associated with dysbiosis in diabetes knock-out mice [95]. Another study showed that resveratrol treatment reduced the proportion of harmful bacteria such as *Desulfovibrio* and *Lachnospiraceae* in the guts of animals with hepatic steatosis [96], and *Bilophila* and *Ruminococcus* in the guts of animals with high fat diet-induced metabolic imbalance conditions [97]. Other studies mention that resveratrol helps the gut to maintain the gut barrier integrity and function and inhibits inflammation in the gut [98,99] (Figure 2).

Based on these findings, we assume that resveratrol could help to reduce harmful gut microbes in patients with HE, thus supporting the suggestion that resveratrol has therapeutic potential for HE and that its therapeutic effect might be mediated by improvements in the gut microbiome and gut barrier function following metabolic imbalance.

## 4. Resveratrol and Brain Edema in HE

Brain edema is defined as an excessive accumulation of water in the intra- and extracellular spaces in the brain and is a common feature in HE [100]. Vasogenic edema leads to the breakdown of the BBB via the loss of tight junction proteins and raises the intracranial pressure [101], while cytosolic edema increases BBB permeability via the intracellular swelling of the astrocytes and also increases brain volume [102]. Cytotoxic edema triggers an increase in the water permeability of the cell membrane via changes in the expression of water transport membrane proteins such as aquaporins [103]. This is especially true for aquaporin 4 (AQP4), which is the most common water channel protein in the CNS and is found in high quantities in both the perivascular area and astrocyte end-feet [103].

Astrocytes function as a component of the BBB and exhibit a higher capacity for water permeability than the other parts of the BBB [104]. Their swelling is commonly associated with the regulation of osmo-intracellular pathways such as calcium signaling and the alteration of aquaporin protein expression in the water transport and ion channels [104,105,106,107]. Increases in the expression of aquaporins, such as AQP4, correlate with the progression of brain edema [108,109]. The majority of HE-associated brain edema cases can be described as cytosolic edema and are closely associated with astrocyte swelling [110,111]. Many acute HE patients demonstrate brain edema accompanied by intracranial pressure increases [110,111], and some studies have linked the excessive brain edema associated with liver failure models to changes in the regulation of the brain tissues [100,112,113].

Therefore, the modulation of the water permeability in HE brains is an important consideration for therapeutic intervention, as this edema often determines the degree of neuropathological damage in these tissues. Resveratrol has been known to ameliorate ischemic brain edema through the inhibition of AQP4 expression [114] (Figure 1A). In addition, resveratrol suppresses cerebral edema by inhibiting the Na^+^ channel-related SUR1 expression in the brain and subsequently influences osmotic cell swelling [115]. Further, SIRT1 activation following the addition of resveratrol decreased BBB breakdown by protecting against the loss of tight junction proteins through improved SIRT1/p53 signaling and ultimately decreased brain edema [116]. Another study showed that the intra-arterial administration of resveratrol exerted a beneficial effect on cerebral ischemic edema in rats [117].

A recent study demonstrated that resveratrol could alleviate astrocyte swelling in response to ammonia-induced oxidative stress [118]. In this study, however, it was also shown that resveratrol can enhance ammonia-induced cell swelling under certain concentrations. Thus, caution is recommended when resveratrol is used for the treatment of the neurological conditions associated with brain edema. Therefore, more studies are required to confirm the beneficial role of resveratrol on brain edema.

Matrix metalloproteinase (MMP) is a key extracellular matrix component responsible for the maintenance of the BBB [119,120]. Resveratrol suppresses the increased expression of MMP linked to the occurrence of cerebral edema events [121]. Resveratrol was also shown to maintain BBB integrity by controlling the MMP-9/TIMP-1 balance after cerebral ischemia-reperfusion in rats [122]. This protective effect was then confirmed again in an additional study using an animal model of cerebral ischemic injury [123]. It was also reported that resveratrol reduces the level of MMP-9 in the BBB and blocks BBB disruption [124,125]. In addition, resveratrol pre-treatment improved BBB breakdown via its interactions with the YAP/TAZ signaling pathway in the brain [126], Moreover, resveratrol protected BBB integrity and improved cognitive function in AD rats [127]. A recent study demonstrated that resveratrol restores the tight junction protein expression in the BBB and helps to inhibit severe neuropathology in response to hyperammonemia in mice with liver cirrhosis [128].

Given these data, we surmise that resveratrol may prevent brain edema by protecting the BBB and facilitating the maintenance of its integrity. Although more studies are required to prove the safety of using resveratrol for the treatment of brain edema, many studies emphasize the fact that resveratrol could be used as an inhibitor of brain edema in response to HE.

## 5. Resveratrol and Ammonia-Induced Neuroinflammation in HE

Ammonia (NH_3_ and NH_4_^+^) is a critical factor in several important cellular functions in the CNS, including the secretion of excitatory and inhibitory neurotransmitters [129], mitochondrial permeability [130,131], ion homeostasis [132], inflammatory responses [133], and oxidative stress [134,135]. Ammonia is the critical factor in the development of HE, and a high level of these ions (hyperammonemia) leads to astrocyte swelling [107], BBB breakdown [136], high levels of ROS [137], neuronal cell death [138], energy deficits [139], glutamine synthetase inactivity [132,140], nitrogen species production [141], and impaired cognition [142]. The redox imbalance caused by a high level of ammonia induces the oxidation of many biomolecules and the inactivation of the antioxidant enzymes [141]. In HE, hyperammonemia triggers oxidative stress and the excessive generation of ROS, inducing various neuroinflammatory responses [143,144], accelerating the activation of the microglia and astrocytes, and amplifying neuroinflammation [142,145,146] (Figure 1B).

Resveratrol has been shown to alleviate the brain damage associated with hepatic ischemic stress by decreasing the activity of aminotransferase [147] and reducing the expression of interleukin (IL)-1 beta and IL-6 [148]. Recent studies also suggest that a supplementation with resveratrol inhibits mitochondrial dysfunction in response to increased levels of ammonia and improves cellular redox in astrocytes affected by ammonia-mediated toxicity [149,150]. Another study showed that resveratrol suppresses ammonia levels in the brain and prevents the severe exacerbation of HE from increased liver cirrhosis [128]. Some studies mention that resveratrol inhibits DNA damage in the neurons and protects against cell death in response to ammonia toxicity [151,152]. Another study suggested that resveratrol promotes DNA repair in response to oxidative stress [153].

Additionally, resveratrol treatment improved the antioxidant capacity and induced mitochondrial biogenesis during oxidative stress [154]. Resveratrol can also prevent neuronal damage during ammonia-induced oxidative stress [8]. Given these data, we can conclude that resveratrol has strong potential as a treatment for HE and that this therapeutic effect is likely mediated, at least in some part, by its inhibition of the inflammatory response and the inhibition of neuronal damage.

## 6. Conclusions

Here, we reviewed the therapeutic potential of resveratrol in the treatment of HE, focusing on gut microbiota, brain edema, and neuroinflammation. Our review supports the assertion that resveratrol is beneficial in reducing harmful gut microbes and maintaining the gut barrier integrity in response to metabolic imbalances. It also demonstrates that resveratrol reduces brain edema via the regulation of both water permeability in the BBB and astrocyte swelling. Finally, these data clearly show that resveratrol alleviates neuroinflammatory responses by activating antioxidant enzymes and inhibiting DNA damage in response to oxidative stress. Given the fact that previous clinical studies have tried to use resveratrol supplementation as a treatment in liver injury [155,156,157], we also emphasize the therapeutic potential of resveratrol in liver failure and suggest that resveratrol therapy may be a promising clinical approach for HE. A recent study demonstrating the improvement in the neuropathology of HE patients treated with resveratrol further supports these observations [158].

However, we also note that there are still debates on the use of resveratrol for therapeutic purposes. One of the issues is the low bioavailability of resveratrol [159]. Moreover, there is another issue related to ammonia-induced cell swelling under certain concentrations, as described above [118]. Moreover, there were meta-analyses suggesting that the effect of resveratrol on the cognitive effects of the human brain may be limited [35,36]. Although more studies are required to scrutinize these points, resveratrol is still expected to be a promising candidate for the treatment of HE, according to its diverse effects related to HE described in this review.

Taken together, this review emphasizes that resveratrol has multiple therapeutic potentials for the treatment of HE. Since the effects and mechanisms of resveratrol in HE patients are not fully elucidated, further studies need to be undertaken to help explain the specific mechanism underlying the therapeutic effects of resveratrol in HE patients. Given that resveratrol is a natural compound, we suggest that resveratrol may be a promising agent with fewer side effects for the treatment of HE.
